# Decreased expression of ferroportin in prostate cancer

**DOI:** 10.3892/ol.2021.12518

**Published:** 2021-02-04

**Authors:** Dong Xue, Cui-Xing Zhou, Yun-Bo Shi, Hao Lu, Xiao-Zhou He

Oncol Lett 10: 913-916, 2015; DOI: 10.3892/ol.2015.3363

Following the publication of the above article, an interested reader drew to the authors’ attention that, in Fig. 1 on p. 914, the data panels selected for Fig 1C and D showing the expression of ferroportin protein in tissue samples for moderately differentiated and poorly differentiated prostate cancer, respectively, contained overlapping information and were ultimately derived from the same original source. The authors re-examined their original data, and realized that the wrong data were selected for Fig. 1D.

The corrected version of Fig. 1, featuring the correct data panel for Fig. 1D (showing poorly-differentiated prostate cancer), is shown opposite. Note that the correction made to this figure does not affect the overall conclusions reported in the paper. The authors are grateful to the Editor of *Oncology Letters* for allowing them the opportunity to publish this corrigendum, and apologize to the readership for any inconvenience caused.

## Figures and Tables

**Figure 1.**
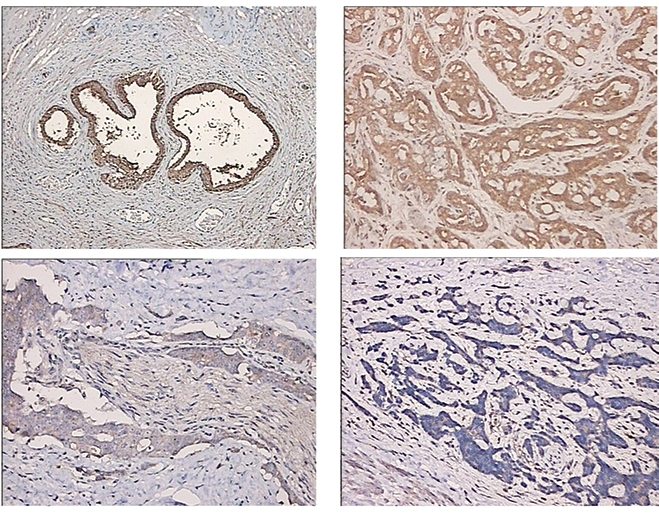
Expression of ferroportin protein in tissue samples, as determined by immunohistochemistry. (A) Benign prostatic hyperplasia tissue samples; (B) highly-differentiated prostate cancer (Gleason score, <7); (C) moderately-differentiated prostate cancer (Gleason score, 7); and (D) poorly-differentiated prostate cancer (Gleason score, >7) (×200 magnification).

